# Supplementary material for: Fabrication of Cell-Loaded Two-Phase 3D Constructs for Tissue Engineering
Source: Materials (Basel). 2016 Nov 1;9(11):887. doi: 10.3390/ma9110887 (PMC5457208; doi:10.3390/ma9110887)
Supplement: Supplementary file 1 [file materials-09-00887-s001.pdf]

Supplementary Materials: Fabrication of Cell-Loaded Two-Phase 3D Constructs for Tissue Engineering

Tobias Zehnder, Tim Freund, Merve Demir, Rainer Detsch and Aldo R. Boccaccini

Wettability of Scaffolds—Effect of PEG Blending

In Figure S1 the effect on the wettability of the scaffolds by blending PCL with 30 wt. % PEG is presented. The pure PCL scaffold is swimming on the surface as the deionised water cannot infiltrate the porous structure and so the remaining air causes buoyancy forces. The scaffold blended with 30 wt. % PEG is infiltrated and the scaffold sinks to the ground.

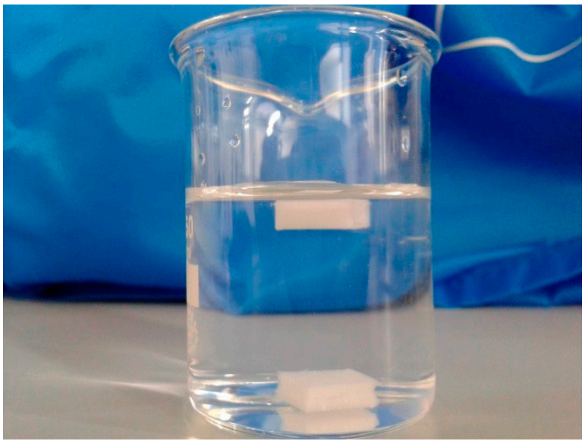

Figure S1. Effect of PCL blending with PEG on the wettability of plotted scaffolds.

Biocompatibility of the Sequential Bioplotting Process—Influence of the Thermoplastic Processing Temperature on the Cell Viability at the Interface between PCL-PEG and ADA-GEL

The influence of the elevated plotting temperature (85 °C) of a PCL-PEG 8020 blend on the viability of ST2 cells mixed in an ADA-GEL hydrogel was evaluated. At first a layer with the thermoplastic phase followed by a layer of the hydrogel on top of it was plotted to simulate the possibly "harshest" conditions for the cells on the interface between the two materials. In Figure S2a,b an optical image and a thermogravic camera image is presented. These images indicate the rapid cooling of the thermoplastic phase after it is released from the cartridge. In Figure S2c,d the same image combination is presented for the hydrogel phase, which processing temperature was set to 37 °C.

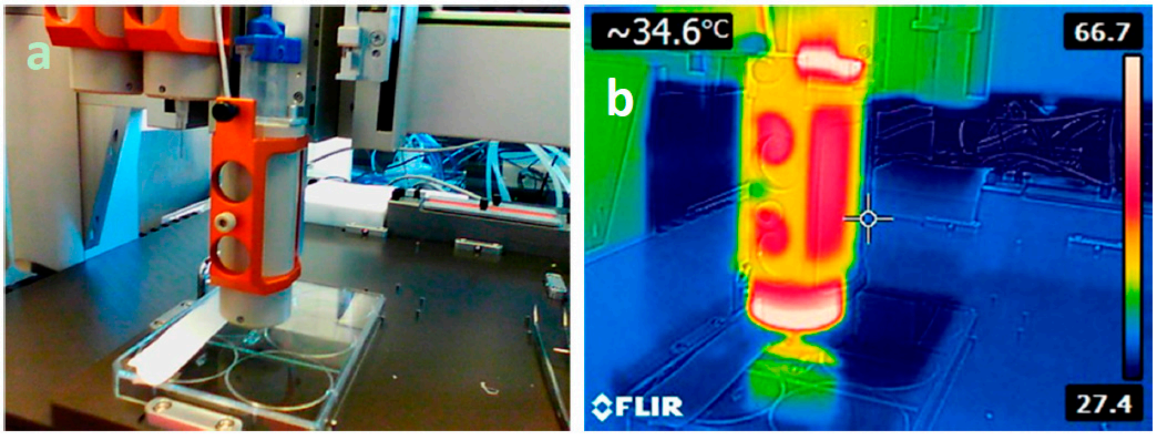

Figure S2. *Cont.*

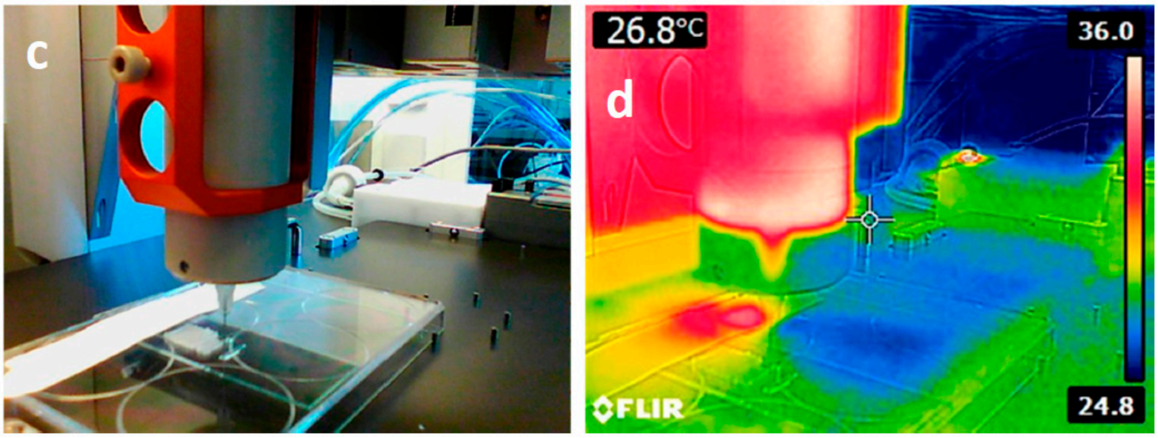

Figure S2. Optical and thermogravic camera images of the plotting of PCL-PEG 8020 (**a**,**b**) at 85 °C and ADA-GEL hydrogel at 37 °C (**c**,**d**) on top of each other.

In Figure S3a the percentage of viable cells on total number of of ST2 cells plotted into a wellplate (=reference) and plotted on a before processed thermoplastic phase. In Figure S3, (b) (reference), (c) (thermoplastic interface) representative images of the live (green)–dead (red) staining are shown. There was no significant difference between the two groups indicating the biocompatibility of the sequential bioplotting process.

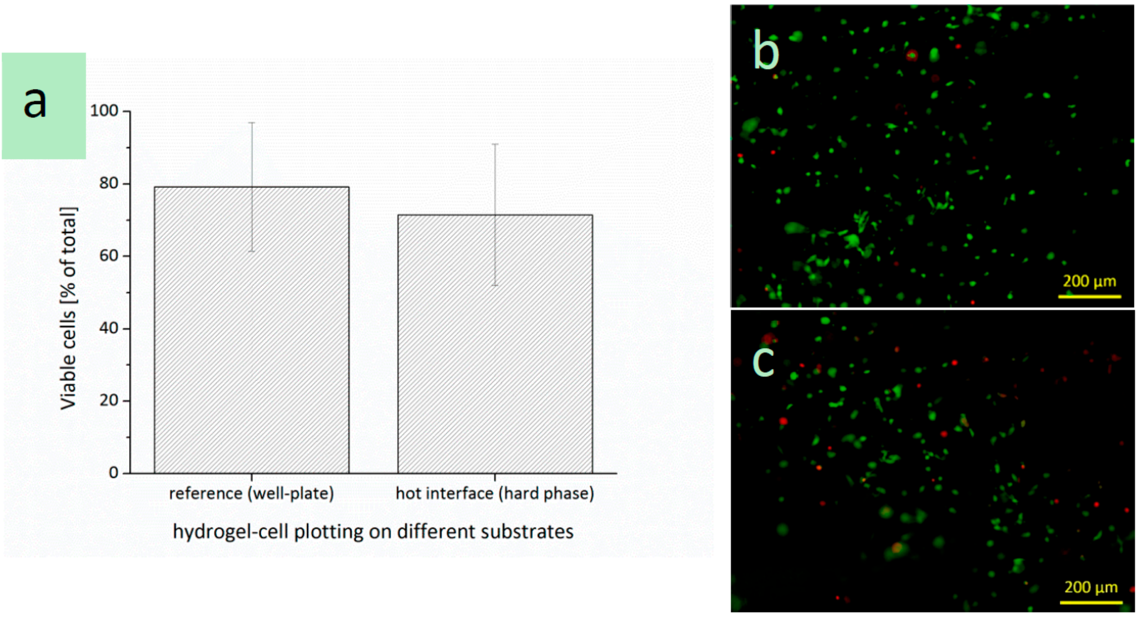

Figure S3. Percentage of viable cells on total cell number (**a**) and representative images of the live-dead staining of ST2 cells in ADA-GEL plotted into well plate (=reference) (**b**) and plotted on hot thermoplastic interface (**c**) to evaluate influence of the processing temperature on the cell viability.
